# Meeting of the American Medical Association

**Published:** 1858-07

**Authors:** 


### PROCEEDINGS OF SOCIETIES.

### ARTICLE VII.

*Meeting of the American Medical Association.*

THE Association held its eleventh annual meeting, this year, in the City of Washington. The number of delegates present was very large—about five hundred. As usual, there was much harmony of sentiment, and the strong determination manifested to uphold the ethics of the profession was extremely cheering. If any one who was present ever entertained doubts of the moral force of the Association, such doubts must have been dissipated by what there occurred. The meeting was in all respects a most satisfactory and delightful one. Receptions were given to the Association by the President of the United States and by Judge Douglas, and by several members of the profession.

The last day was devoted to an excursion to Mount Vernon, and was, we are told, a most delightful one.

All that the most liberal and elegant hospitality could furnish, was provided by the brethren of Washington for the entertainment and gratification of their guests, and we are confident that the meeting in Washington will be admitted by all to be among the most delightful ever held.

We subjoin an abstract of the business proceedings of the Association :

Tuesday Morning, May 4th, 1858.—The Association assembled in the beautiful and commodious lecture room of the Smithsonian Institution, at 11 o'clock, A. M., when the chair was taken by the President, Dr. Eve, of Nashville.

Dr. R. C. Foster, of Tennessee, and A. J. Semmes, of Washington, Secretaries, were present.

The chairman of the Committee of Arrangements, Dr. Harvey Lindsley, welcomed the members in a brief and pertinent address.

The names of the delegates who had registered were reported by the Committee of Arrangements, and the roll was then called by the Secretary. Twenty-five States, the District of Columbia, the Medical Staff of the U. S. Navy, and the American Medical Society in Paris were represented.

Dr. Lindsley, chairman of the Committee of Arrangements, reported that it was proposed the Association should hold but one business session each day, from 9, A. M. until 3, P. M.; which proposal was on motion adopted. He also announced that the President of the United States would be happy to receive those members of the Association who might call at the Executive Mansion at 8 o'clock in the evening, and such ladies as may accompany them. He further stated that there had occurred a vacancy in the Committee of Arrangements, which had been temporarily filled by the appointment of Dr. J. M. Snyder, and he asked the Association to confirm this act, which was on motion adopted.

A recess of fifteen minutes was then ordered, to enable the different State delegations to nominate one of their number to constitute the Committee of Nominations. A question having been started as to the right of the army and navy delegations to be represented in this committee, the chair decided in their favor, and this decision was sustained by the Association.

On re-assembling, the following gentlemen were reported by the delegations as having been selected by them for the Committee of Nominations :

*Maine*—Job Holmes.
*New Hampshire*—Geo. H. Hubbard.
*Vermont*—P. Pineo.
*Massachusetts*—Ebenezer Alden.
*Connecticut*—Ashbel Woodward.
*Rhode Island*—J. Mauran.

*New York*—H. D. Bulkley.
*New Jersey*—J. P. Coleman.
*Pennsylvania*—Isaac Hays.
*Delaware*—H. F. Askew.
*Maryland*—S. P. Smith.
*District of Columbia*—Noble Young.
*Virginia*—A. S. Payne.
*North Carolina*—W. H. McKee.
*South Carolina*—Wm. T. Wragg.
*Georgia*—Joseph P. Logan.
*Alabama*—J. T. Hargraves.
*Kentucky*—R. J. Breckinridge.
*Tennessee*—J. Berrian Lindsley.
*Missouri*—Wm. M. McPheeters.
*Ohio*—George Mendenhall.
*Indiana*—Calvin West.
*Illinois*—A. H. Luce.
*Michigan*—Zina Pitcher.
*Iowa*—Thomas O. Edwards.
*California*—O. Harvey.
*United States Navy*—George Clymer.

On motion, Drs. Bohrer, of D. C., A. Flint, of New York, and J. T. Hargraves, of Alabama, were appointed by the President a Committee on Special Essays.

The Chair announced that Dr. D. Meredith Reese, of New York, desired permission to make a communication, which having been granted, Dr. Reese read the following statement:

"*To the Officers and Members of the*
*American Medical Association*:

"The undersigned, one of the Vice-Presidents of the American Medical Association, having, during the interval since our last annual meeting, certified to the professional fitness for the charge of the Blockley Hospital, at Philadelphia, of an individual who had been expelled from this body for a violation of our code of ethics, after consultation with the other officers, and yielding to the advice of other personal friends, desires to say to the Association now assembled—

"1st. That, in giving said certificate, he was prompted solely by motives of sympathy and humanity to a fallen brother, who had been a personal friend prior to his offence; and that he did not realize, acting under the impulse of the moment, that his individual act could be construed by the profession as indicating hostility to his brethren.

"2d. That while his own mind is clear that his certificate contained only the truth, and that, under his peculiar relations to the party concerned, he could not withhold his certificate of medical qualification consistent with conscience and duty, yet he is ready to concede that he had no abstract right to relieve the party from the censure of the Association until this body had restored him to its fellowship.

"3d. That, so far from intending any disrespect to the Association, or to its act of discipline, the undersigned had publicly sustained and defended both. He therefore disclaims the inference from his certificate that he intended to recommend to a high professional office, a man whom the Association had excluded, and thereby nullify the action of this body.

"And, finally, with these statements and disclaimers, the undersigned, while retaining his own opinion of the rectitude of his motives, and of his duty, under the peculiar circumstances of the case, is nevertheless prepared to defer to the judgment of those whom he knows to be his friends, that he erred in doing what he had no right to do, in view of his official position in the Association, and is hence called upon to offer this explanation and apology to his brethren.

David M. Reese."

Dr. Condie, of Pennsylvania, moved that the apology be received.

An amendment to this motion, declaring that the Code of Ethics of the Association had been violated, was offered. The amendment was not agreed to; and the original motion of Dr. Condie was adopted.

On motion of Dr. Atlee, of Pennsylvania, the statement of Dr. Reese was ordered to be entered upon the minutes.

Dr. James Bryan, of Pennsylvania, said that he was in the same position with Dr. Reese, and offered a similar statement, which was, on motion of Dr. Condie, of Pennsylvania, received.

On motion of Dr. Lindsley, of D. C., Dr. Paul F. Eve, of Tennessee, the retiring President, delivered the annual address.

On motion of Dr. Atlee, of Pennsylvania, the President was requested to furnish a copy of his address for publication in the *Transactions* of the Association.

Reports from Standing Committees being in order, Dr. Grafton Tyler, of D. C., Chairman of the Committee on Prize Essays, submitted the following report:

"The Committee on Prize Essays report, that the Essays received were three in number, each of which has been examined by them with great care, considering, first, the intrinsic merits of each essay, and then their merits in relation to each other.

The Committee have discharged the responsible and delicate duty imposed upon them, with a consciousness of its great importance, and are gratified to be able to declare two of the essays submitted to their consideration, in their judgment, each worthy of a prize. The third they also highly commend.

The Committee award the first prize to the essay entitled "An Essay on the Clinical Study of the Heart Sounds, in Health and Disease." It bears the motto, "*Clinica clinice demonstrandum.*"

They award the second prize to the essay entitled "An Essay on Vision, and some of its Anomalies, as revealed by the Ophthalmoscope." It bears the motto, "*Dux Hominum, medicus est.*"

GRAFTON TYLER,
J. C. HALL,
JNO. FRED. MAY,
THOMAS MILLER,
JOSHUA RILEY,
W. J. C. DUHAMEL,
A. J. SEMMES,
*Committee on Prize Essays.*

On motion, the report was accepted, and the essays were referred to the Committe on Publication. Dr. G. Tyler, then, in the presence of the Association, opened the sealed packets, disclosing the names of the authors, and found the "Essay on the Clinical Study of the Heart Sounds, in Health and Disease," to which was awarded the first prize, to have been written by Dr. Austin Flint, of Buffalo, N. Y.; and that the "Essay on Vision, and some of its Anomalies, as revealed by the Ophthalmoscope," to which was awarded the second prize, to have been written by Dr. Montrose A. Pallen, of St. Louis, Missouri.

Upon the motion of Dr. Palmer, of Michigan, and by general consent, Drs. Flint and Pallen each favored the Association with a brief synopsis of their essays.

Dr. Lindsley, from the Committee of Arrangements, presented a letter from Capt. M. C. Meigs, U. S. Engineers, calling the attention of the Association to the subject of ventilation, and inviting the members to visit the capitol extension, with a view to its examination, in this connection; also, a letter from Dr. C. H. Nichols, in charge of the U. S. Hospital for the Insane of the Army and Navy, and of the District of Columbia, inviting the members to visit that establishment; and a communication from the Rev. Bernard A. Maguire, President of Georgetown College, inviting the Association to visit the Institution.

On motion of Dr. Hammond, of New York, these several invitations were accepted, and the thanks of the Association were accorded therefor.

On motion of Dr. H. Lindsley, Chairman of the Committee of Arrangements—

The Hon. Drs. Fitch, of Indiana, Chaffee, of Massachusetts, Clawson and Robbins, of New Jersey, and Shaw, of North Carolina, members of Congress, and Dr. Peter Parker, ex-Commissioner to China, and H. Dean, of Maryland, were elected "members by invitation," and requested to participate in the proceedings of the Association.

On motion, Assistant Surgeon Frederick A. Rose, of the British Navy, who so nobly volunteered his services on board the United States Ship Susquehanna, at Port Royal, and who came in her to New York, devoting himself to the sick crew, was unanimously elected a "member by invitation," and invited to take a seat upon the platform. [It was supposed at the time that Dr. Rose was in the city, but it was ascertained that he had left.]

Dr. Francis G. Smith, of Philadelphia, Chairman of the Committee on Publication, made his report, which was accepted, and referred to the Committee on Publication.

Dr. Caspar Wister, of Philadelphia, Treasurer, presented his annual report, accompanied with the following resolutions, the adoption of which he recommended:

*Resolved*, That on or after any annual meeting of this Association, each of the back numbers of *Transactions* shall be sold at $2 a piece to permanent members, except that published during the year next preceding—this to continue at the price paid for it by delegates till the next annual meeting; and also

*Resolved*, That as certain volumes are in great excess, the four volumes (v. vii. viii. ix.) shall be sold collectively to any permanent member who shall remit $5 to the Treasurer.

The report was accepted, and referred to the Committee on Publication, and the resolutions were adopted.

The Special Committee on Medical Education, of which Dr. G. W. Norris, of Philadelphia, is Chairman, were called upon to report. There was no response; and on motion, the subject was referred to the Committee on Nominations.

Dr. A. B. Palmer, Chairman of the Committee on Medical Literature, asked leave to defer his report until Wednesday, at 10 o'clock ; which was granted.

The Committee of Nominations, through their Chairman, reported the following for officers for the ensuing year, which report was adopted.

*President.*—Dr. Harvey Lindsley, of Washington City.

*Vice-Presidents.*—Drs. W. L. Sutton, of Kentucky ; Thomas O. Edwards, of Iowa ; Josiah Crosby, of New Hampshire ; and W. C. Warren, of North Carolina.

*Secretary.*—Dr. A. J. Semmes, of Washington City. [The other Secretary will be nominated when the location of the next meeting of the Association is selected.]

*Treasurer.*—Dr. Caspar Wister, of Philadelphia.

On motion, Drs. Flint, of New York, Gross, of Pennsylvania, and Gibbes, of South Carolina, were appointed a committee to conduct the newly-elected officers to their seats.

Dr. Lindsley, having been introduced to the Association by the retiring President, made a few pertinent remarks, acknowledging the honor as the highest he had ever been called upon to receive, and the highest that any medical man in America can receive.

On motion, the thanks of the Association were unanimously accorded to the retiring officers, for the able manner in which they had performed their duties ; and they were also invited to take seats upon the other platform.

On motion, the Ex-Presidents of the Association present were invited to take seats on the platform.

On motion of Dr. Palmer, of Michigan, it was ordered that the Special Committee on Medical Education (Dr. Jas. R. Wood, of New York,) report Wednesday morning, immediately after the report on Medical Literature.

Next in order were reports from the Committees on Medical Topography and Epidemics, from each of the several States of the Union. The report from Maine was referred to the Committee on Nominations.

New Jersey being called, Dr. Lyndon A. Smith, Chairman, of the Committee, read extracts from his report, which was referred to Committee on Publication.

The Association then, on motion, adjourned until Wednesday morning, at 9 o'clock.

---

Wednesday, May 5.—The Association was called to order at 9 o'clock, by the President, Dr. Lindsley.

The minutes of the first day's proceeding were read by Dr. A. J. Semmes, one of the Secretaries, and adopted.

On motion of Dr. Watson, of New York, Dr. Delafield, of New York, one of the first officers of the Association, was invited to take a seat on the platform.

Dr. Thos. P. Atkinson, of Virginia, submitted the following:

*Resolved,* That the Constitution of this Association be so amended as to provide that no individual who shall be under sentence of expulsion or suspension, from any state or local medical society of which he may have been a member, shall be received as a delegate to this body, or be allowed any of the privileges of a member until he shall have been relieved from said sentence by such state or local society.

The President decided that under the Constitution, amendments to it must lie over one year, and the resolution was accordingly laid on the table, for consideration at the next annual meeting.

On motion of Dr. Paul F. Eve, of Tennessee, Dr. William M. Cumming, of Georgia, for some time missionary physician to China, was elected a member by invitation.

Drs. Huff and Knight were proposed by the Committee of Arrangements as members by invitation, and the same was adopted.

Dr. A. B. Palmer, of Michigan chairman of the Committee on Medical Literature, presented a report.

The report was accepted and referred to the Committee on Publication for insertion in the *Transactions.*

On motion of Dr. Gross, Dr. Bozeman, of Alabama, was elected a member by invitation.

Dr. James R. Wood, chairman of a Special Committee on Medical Education, made a report, discussing 1st, primary medical schools ; 2d, the number of professorships in medical colleges ; 3d, the length and number of terms during the year ; 4th, the requisite qualifications for graduation ; 5th, such other subjects of a general character as to give uniformity to our medical system. Having reviewed these propositions at length, the Committee, he stated, have arrived at the following conclusions :

1st. Primary medical schools should be encouraged ; but, as office instruction will continue to be sought by students, practitioners should either give them necessary advantages of demonstrations, illustrations, and recitations, or if not prepared to do so, they should refer them to such primary schools, or medical men, as will give them proper instruction.

2d. The number of professorships should not be less than seven, viz. a Professor of Anatomy and Microscopy, Physiology and Pathology, Chemistry, Surgery, Practical Medicine, Obstetrics, and Materia Medica.

3d. There should be but one term annually, which should commence about the 1st of October, and close with the March following, thus lengthening the term to six months. The commencement of the term, in October, should be uniform in all the colleges throughout the country. During the session there should never be more than four lectures given daily.

4th. The qualifications for graduation, in addition to those now required by the schools, should be a liberal primary education, and attendance upon a course of clinical instruction in a regularly organized hospital.

In order to give our medical colleges an opportunity to consider the recommendations here advanced, and that this body may have the advantage of their wisdom and their mature views, before any definite action is taken upon them, your Committee submit to the Association the following resolutions:

*Resolved*, That the several medical colleges of the United States be requested to send delegates to a convention to be held at on the day of for the purpose of devising a uniform system of medical education.

*Resolved*, That the present report of the Special Committee on Medical Education be referred to such convention for its consideration.

*Resolved*, That said convention of delegates from the several colleges of the United States be requested to submit to the meeting of this Association in May, 1859, the result of their deliberations.

On motion, the report was accepted and referred to the Committee on Publication, the accompanying resolutions being laid on the table.

The Committee of Nominations made a further report, recommending Louisville, Ky., as the place of meeting in 1859, and nominating Dr. S. S. Bemis, of that city, as second Secretary. They also nominated the following standing committees:

*Committee on Publication.*—Dr. F. G. Smith, Pa., Chairman; Drs. Caspar Wister, Pa.; A. J. Semmes, D. C.; S. M. Bemis, Ky.; S. L. Hollingsworth, Pa.; S. Lewis, Pa.; H. F. Askew, Del.

*Committee on Medical Literature.*—Dr. John Watson, N. Y., Chairman; Drs. L. A. Smith, N. J.; C. G. Comegys, Ohio; R. W. Gibbs, S. C.; W. M. McPheeters, Mo.

*Committee on Prize Essays.*—Dr. J. B. Flint, N. Y., Chairman; Drs. M. Goldsmith, Ky.; H. Miller, Ky.; Calvin West, Ind.

*Committee on Medical Education.*—Dr. G. W. Norris, Pa., Chairman; Drs. A. H. Luce, Ill.; E. R. Henderson, S. C.; G. R. Grant, Tenn.; T. S. Powell, Ga.

*Committee of Arrangements.*—R. J. Breckinridge, Ky., Chairman; Drs. G. W. Ronald, B. M. Wible, D. W. Yandall, D. D. Thompson, N. B. Marshall, G. W. Burglass, R. C. Hewitt, and A. B. Cook, all of Kentucky.

The report was accepted, the nominations were confirmed and the Committee received permission to sit again.

On motion, the resolutions attached to the report of the Committee on Medical Education were taken from the table for consideration.

After considerable discussion, it was, on motion of Dr. Hamilton, of New York, resolved to refer the resolutions to a committee, consisting of one member from each delegation representing a medical college, these members to be named by the college delegates present, and to report on Thursday morning at 10 o'clock.

On motion, the thanks of the Association were voted to the retiring Secretary, Dr. Foster, for his valuable services, and his successor, Dr. Bemis, was inducted into his seat.

On motion of Dr. J. F. Lamb, of Pennsylvania, the rules were suspended for the purpose of reconsidering the resolution adopted the previous day, to accept the apology of Dr. D. Meredith Reese, of New York ; yeas 142, nays 70.

After considerable debate, it was, on motion, resolved that the Association go into Committec of the whole, and Dr. Edwards, of Ohio, was called to the chair.

A memorial from the Philadelphia County Medical Society calling the attention of the Association to the fact of Drs. Reese and Bryan, recommending Dr. McClintock as Physician-in-Chief of the Blockley Hospital, was presented by a committee of the above mentioned Society, and read.

On motion of Dr. Humphreys, of Indiana, it was resolved, that each member of the Committee of the Whole be restricted to five minutes, allowing Dr. Reese whatever time he wished to defend himself in.

Dr. Reese then made a statement of his position from the commencement of the controversy, and stated that he had intended the apology made the previous day to be a satisfactory one, and that if it was not such, he would make one that should be satisfactory.

After considerable discussion, in the course of which Dr. Reese stated that he wished his apology to be taken as full, and without mental reservation, the Committee of the Whole rose, and the Chairman reported to the President that the Committee had heard and discussed the apology of Dr. Reese, and that they considered that it was "ample, full, complete and satisfactory."

On motion, the report of the Committee was received and adopted.

The case of Dr. Bryan then came up, when it was suggested that the amended apologies of Drs. Reese and Bryan should be in writing, the latter expressing a willingness to make one similar to that of Dr. Reese.

Dr. Reese then drafted an apology, which was not, however, considered satisfactory, as not expressing his *regret* for what he had done, and after several attempts, which gave rise to an exciting debate, he finally presented the following, which was accepted as satisfactory:

"The undersigned regrets that he certified to the professional qualifications for Blockley Hospital, Philadelphia, of an expelled member of this body, and hereby offers this apology for his departure from the ethical code.

Signed,

D. Meredith Reese."

Dr. Bryan, who had successively offered to adopt the several apologies as presented by Dr. Reese, but refusing to go further, finally consented to adopt the one last offered by Dr. Reese. After Dr. Bryan had gone to the Secretaries' table and affixed his signature to the apology, in compliance with the demand of the meeting, his apology was accepted.

The President announced that the Faculty of Georgetown College had invited the Association to an entertainment at the college this afternoon, and that omnibuses would leave the principal hotels at 5 o'clock, to convey the members thither.

The Association then adjourned to Thursday morning, at 9 A. M.

Thursday, May 6, 1858.—The President called the Association to order at 9 o'clock. The reading of the minutes of yesterday was temporarily postponed.

The following resolution, offered by Dr. R. C. Foster, of Tennessee, was adopted :

*Resolved,* That all reports from Committees on Epidemics and Medical Topography, be referred to the Committee on Publication, and all communications from Special Committees be referred to the Committee of Nominations.

Dr. Paul F. Eve, of Tennessee, moved that a Committee on Voluntary Essays be appointed; also, that Dr. Geo. Hayward, of Massachusetts, be appointed a delegate to represent this Association in kindred societies in Europe, which were agreed to.

An invitation was received and accepted, from Commander M. P. Maury, United States Navy, Superintendent of the National Observatory, inviting the Association to visit that establishment.

Dr. Hamilton, of New York, from the Committee of Delegates from Medical Schools and Colleges, to whom was referred the report of the Special Committee on Medical Colleges, reported the following preamble and resolution :

Fully appreciating the value and importance of the resolution under which they were appointed, but a majority of the gentleman constituting this committee, not being authorized by the medical faculties of the several colleges with which we are connected to act as their representatives in this matter, and, therefore, regarding it quite impossible to secure a convention of delegates in the interim of the meetings of the Association : therefore—

*Resolved,* That we recommend to all the medical colleges entitled to a representation in this body, that they appoint delegates, especially instructed to represent them in a meeting to be held at Louisville on Monday, the day immediately preceding the convention of the American Medical Association for the year 1859, at 10 o'clock, at such place as the Committee of Arrangements shall designate.

The report was accepted and the preamble and resolutions adopted.

The reports of committees being decided to be in order,

Dr. Foster Jenkins, New York, read a report on the spontaneous umbilical hemorrhage of the newly born; which was read and referred to the Committee on Publication.

Dr. S. M. Bemis, of Kentucky, read a report on the "Influence of Marriages of Consanguinity upon Offspring," which was referred to the Committee on Publication.

Dr. J. L. Atlee, from the Committee appointed to procure a stone to be inserted in the Washington Monument, made a final report.

A report by Dr. E. Andrews, of Chicago, on the functions of the different portions of the cerebellum, was presented, and referred to the Committee on Publication.

Dr. H. F. Campbell, of Georgia, read a report on the "Nervous Concomitants of Febrile Diseases," which was accepted and referred to the same Committee.

Dr. J. Marion Sims, of New York City, read an abstract of his report on the treatment of the results of obstructed labor, illustrated with a series of illustrations, which was referred to the same Committee.

Dr. M. Stephenson, of New York, read an abstract of his report on "the treatment best adapted to each variety of cataract, with the method of operation, place of selection, time, age, &c.;" which was referred to the same Committee.

Dr. C. B. Coventry, of New York, Chairman of the Committee on the "Medical Jurisprudence of Insanity, and the testimony of skilled witnesses in the Courts of Justice," communicated his report, which was accepted and referred to the Committee on Publication.

Dr. Edwards, from the Committee of Nomination, presented the following list of Committees for the ensuing year which report was adopted.

*Special Committee on the Microscope.*—Drs. Holston, of Ohio, Dalton, of New York, Hutchinson, of Indiana, Stout, of California, and Ellis, of Massachusetts.

*Special Committee on Medical Jurisprudence.*—Drs. Stephen Smith, of New York, Hamilton, of Buffalo, Crosby, of New Hampshire, Purple, of New York, and Mulford, of New Jersey.

*Committee on Quarantine.*—Drs. Harris, of New York, Moriarty, of Massachusetts, La Roche, of Pennsylvania, Wragg, of South Carolina, and Fenner, of Louisiana.

*Committee on Surgical Pathology.*—Dr. James R. Wood, of New York, Chairman.

*Committee on Diseases and Mortality of Boarding Schools.*—Dr. C. P. Mattingly, of Kentucky.

*Committee on the various Surgical Operations for the Relief of Defective Vision.*—Dr. Montrose A. Pallen, of St. Louis, T. G. Cogley, of Indiana.

*Committee on Milk Sickness.*—Dr. Edward A. Murphy, of Indiana.

*On the Blood Corpuscle.*—Dr. A. Sager, of Michigan.

*Committee on Medical Ethics.*—Drs. John Watson, of New York, Dalton, of Massachusetts, Emerson, of Pennsylvania, Hamilton, of New York, and Gaillard of South Carolina.

*On the Pons Varolii, Medulla Oblongata, and Spinal Marrow; their Pathology and Therapeutics.*—Dr. S. B. Richardson, Louisville, Kentucky.

*On American Medical Necrology—the Hygienic relations of air, food and water, the natural and artificial causes of their impurity, and the best methods by which they can be made most effectually to contribute to the public health.*—Dr. C. C. Cox, Easton, Maryland.

*On the Effect of Virus of Rattlesnake, &c., when introduced into the System of the Mammalia.*—Dr. A. S. Payne, Paris, Fauquier County, Virginia.

*On the Climate of the Pacific Coast and its Modifying Influences upon Inflammatory Action and Diseases generally.*—Dr. O. Harvey, Placerville, California.

*The Constitutional Origin of Local Diseases, and the Local Cause of Constitutional Diseases.*—Dr. C. F. Heywood, New York.

*Special Committee on Epilepsy.*—Drs. John G. Kyle, Ohio, Chairman; Jno. G. F. Holston, Zanesville, Ohio; D. Cooper Ayres, Green Bay, Wisconsin; H. J. Dunahoe, Sandusky, Ohio; Calvin West, Indiana.

*Causes of the Impulse of the Heart, and the Agencies which influence it in Health and Disease.*—Dr. J. W. Corson, of New York City.

*Best Substitutes for Cinchona and its Preparations in the Treatment of intermittent Fever, &c.*—Dr. B. S. Woodworth, of Fort Wayne, Indiana.

*Special Committee on Government Meteorological Reports.*—Drs. Paul F. Eve, of Tennessee; Zina Pitcher, of Michigan; R. H. Coolidge, United States Army; Thos. Miller, District of Columbia.

*To fill vacancies in the Committee on Medical Topography and Epidemics; and continuations of the Committees.*—James C. Weston, M. D., of Bangor, Maine; Albert Smith, M. D., of Peterborough, New Hampshire; J. Perkins, M. D., of Castleton, Vermont; James H. Dickson, M. D., of Wilmington, North Carolina; Peter C. Gaillard, M. D., of Charleston, South Carolina; Thos. M. Logan, M. D., of Sacramento, California; J. H. Beech, M. D., of Cold Water, Michigan; Charles Hooker, M. D., of New Haven, Connecticut.

Dr. Edwards also reported from the Committee of Nominations the following resolution:

*Resolved*, That a committee of nine be appointed by the Chair to wait on the Hon. Howell Cobb, Secretary of the Treasury, and respectfully to request the restoration of Dr. M. J. Bailey, as Inspector of Drugs and Medicines for the port of New York, at the same time disclaiming all political considerations.

This resolution was, after considerable discussion, adopted, but the vote was subsequently reconsidered, and the consideration of the resolution indefinitely postponed.

Dr. Bohrer, of D. C., Chairman of the Committee on Special Medical Essays, reported that they had not had time to read, much less consider, the papers placed in their hands.

On motion, the Committee was instructed to hand such papers as they deemed worthy to the Committee on Publication.

Dr. Grant, of New Jersey, presented a complaint made by the Newark Medical Society against the Medical College of New York, for a violation of the ethics of the profession. Dr. Edwards presented a similar complaint, and Dr. Oakley a complaint from the Union and Essex County Medical Societies. They were received and referred to the Committee on Ethics.

A communication received from Dr. E. D. Fenner, of Louisiana, was, on motion, referred to the Committee on Publication.

Dr. W. S. Sutton offered the following resolution :

*Resolved*, That a committee of three, to be composed of members belonging to the District of Columbia, be appointed, whose duty it shall be to urge upon the Census Bureau, the great interest which this Association feels in a properly conducted census, and especially that portion relating to vital statistics ; and the importance of securing, at an early day, the services of a physician conversant with vital statistics, to assist in arranging the schedules in that department of the census to be taken in 1860 ; as also to aid in a general supervision over that branch of the census.

This resolution was adopted, and the following gentlemen appointed as the Committee : Drs. Thomas Miller, Thomas Antisell, and A. Y. P. Garnett, all of District of Columbia.

Dr. W. L. Sutton, of Kentucky, also offered the following resolution :

*Resolved*, That a committee of five be appointed, whose duty it shall be to present to the next meeting of this Association, a plan for an uniform registration of births, marriages and deaths, including the nomenclature, as also the classification of diseases, to be adopted in registration reports. And that the report from Dr. E. Jarvis to this meeting be submitted to their use for the balance of this session.

This resolution was also adopted, and the following gentlemen appointed as the committee, viz. Drs. W. L. Sutton, Kentucky; Edward Jarvis, Massachusetts; Edward M. Snow, Rhode Island; Wilson Jewell, Pennsylvania; R. W. Gibbes, South Carolina.

Dr. Kyle, of Ohio, proposed an amendment to the constitution, by which no member can sit as a member or a delegate at meetings of this Association, who is not a graduate of a recognized medical college. Laid over for one year, under the rules.

Dr. L. A. Smith presented resolutions of the New Jersey Medical Society, praying for such changes of the constitution as would establish a board of censors in every judicial circuit of the Supreme Court, who should examine and grant diplomas to all proper members of this Association. Laid over for one year, under the rules.

On motion of Dr. L. Humphreys, of Indiana, the following preamble, resolution and circular were ordered to be incorporated in the *Transactions* of the Association, viz.

Whereas, a circular looking to the interchange of the published Transactions of the various local medical societies of the United States, was unanimously recommended at the last meeting of the Indiana State Medical Society, (a copy of which, as prepared by their committee, is hereto annexed:) and whereas, it is in the power of this Association to promote and successfully carry out the object thereof; therefore,

*Resolved*, That we cordially approve of a general interchange of published Transactions between the various local societies of the United States, and that, to carry out this desirable object, the delegates of the various societies which publish their transactions, be requested to leave the names and post office address of the Secretary thereof, with the Secretary of this Association, which shall be published with its proceedings, and each society is hereby recommended to send by mail, prepaid, *three copies* of their published Transactions, to all of the other societies included in such list.

The Indiana State Medical Society, at its last annual meeting, adopted the following resolution, viz.

*Resolved*, That the President appoint a committee of three to prepare and present to this Society, at its next meeting, a plan for an interchange of published transactions of local societies within the United States; that said committee be authorized and empowered to correspond with the officers of any local society as above stated, and to take such steps generally as may be necessary to accomplish the objects for which this committee is appointed.

The undersigned were appointed said committee. Will you have the goodness to bring this subject to the attention of your Society at its next meeting; ask some definite action, and communicate early the result to this committee?

The benefits of such an interchange of publications, as contemplated by our Society, are numerous and important, as must be obvious to every reflecting mind. We can but barely mention a few of them:

1st. The more general diffusion among our scattered and measurably isolated professional fraternity, of the records of facts, observations and opinions, in a profession abounding, above all others, in them, on a great variety of subjects, and in which, too often, a *few* facts found on record, or observed, are allowed to give direction to conservative or curative efforts of the first importance.

2d. The cultivation of a better acquaintance with professional brethren throughout the country, by means of the minutes of societies, and the papers read before and discussed in them, in a profession affording otherwise but limited opportunities for the formation of such an acquaintance, and therefore needing the more vigorous use of such as are possessed.

3d. The stimulus such an arrangement will afford to the diligent cultivator of the profession, to observe well, to record accurately, speculate philosophically, reason logically, practice usefully, and write perspicuously.

4th. The probable large increase in the number of writers in the profession, and the consequent elevation of the standard of qualification for entering it.

5th. The cultivation of an enlarged and liberal *esprit du corps,* by being brought into contact with the mass of the professional minds of the country.

The Committee solicit suggestions as to the details of a plan for the attainment of this desirable object. They are sensible of many considerable difficulties in the way of it, mainly from want of some channel of intercommunication common to all. Possibly the American Medical Association might become this common channel, and thereby increase its influence.

The *Transactions* might be made the nucleus of a library, where one does not exist in any state or local society, and be used by its members under library regulations. Please acknowledge the receipt of this circular, and communicate at as early a period as possible, the action of your society upon this subject. Hoping to hear that your society concurs in this movement.

Respectfully, yours,

L. Humphreys,<br>

Chas. Fishback, } *Committee.*<br>

R. H. Buck,

Address, Dr. L. Humphreys, (chairman of the committee,) South Bend, St. Joseph County, Indiana.

On motion of Dr. R. W. Gibbes, of South Carolina, it was

*Resolved,* That Prof. G. C. Swallow, of Missouri, and Prof. J. F. Mittag, of South Carolina, be members by invitation of this Association.

On proposition from the Committee of Arrangements, Dr. Blair, of York, Pennsylvania, and Dr. John H. Gibbon, of

United States Mint, North Carolina, were elected members by invitation.

On motion of Dr. H. Fraser Campbell, of Georgia, it was

*Resolved*, That this Association has learned, with deep regret, of the death of the following permanent members of this body: Marshall Hall, England; C. R. Walton, Georgia; S. W. Clanton, Alabama; W. G. Craghead, Virginia; John K. Downes, Connecticut; Nathan S. Pike, Connecticut; W. W. Morris, Delaware; and William Gries, Pennsylvania.

Dr. R. W. Gibbes moved, that Prof. Joseph Henry, of the Smithsonian Institution, be requested to favor this Association with his views on meteorology, at such time during the session as he may select; which was agreed to.

An invitation from Prof. Bache to visit the Coast Survey Bureau, on Capitol Hill, was read, accepted, and a vote of thanks for the courtesy was passed.

On motion of Dr. Phelps, the following resolutions were passed unanimously:

*Resolved*, That the thanks of this Association are eminently due to the Regents and Prof. Henry, of the Smithsonian Institution, for the ample and convenient accommodations afforded for the transaction of business.

*Resolved*, That the Committee of Arrangements are entitled to our praise and highest appreciation of their exertions to promote the comfort of the members, and the best interests of the Association.

*Resolved*, That to the physicians of Washington and Georgetown, and the faculty of Georgetown College, we accord the homage of our sincerest thanks for their elegant hospitalities extended to the members from abroad, by which the pleasure of their sojourn here has been so greatly enhanced.

*Resolved*, That we feel assured that the impressions on the tablet of memory received here, in our national metropolis, in this the first year of the second decade of the Association, will long remain an evidence of the urbane attentions received, not only from the chief magistrate and other public functionaries of our glorious Union, but of private citizens and the community at large.

*Resolved,* That the manifestations of union of heart and purpose in the action of this session, inaugurate a new era, and call for devout acknowledgment to Divine Providence, and presage, as we trust, not only a bright future for the Association, but also as contributing to the perpetuity and prosperity of our great national confederation.

Dr. Arnold, of Georgia, then exhibited specimens of a new method of preserving pathological specimens.

On motion of Dr. Foster, of Tennessee, it was

*Resolved,* That after 1860, Dr. Hamilton have the privilege of using his report on "Deformities after Fracture," published in the *Transactions,* for a work which he proposes to publish.

Dr. Campbell, of Georgia, was not aware, until he had just heard permission granted to Dr. Hamilton, that he had transgressed in republishing in a work a report which he had contributed to the *Transactions* of the Association. [Cries of "Regret it," "regret it."] He did regret it, and asked the sanction of the Society ; which was granted.

Dr. Duhamel, of Washington, moved that a committee be appointed to investigate and report upon the "National Hotel Disease."

Dr. Foster, of Tennessee, opposed the appointment of such a committee, as did Dr. Boyle of Washington. Dr. Duhamel withdrew his motion.

Dr. Gaston, of South Carolina, exhibited and explained a new uterine supporter.

On motion of Dr. Miller, of D. C., it was

*Resolved,* That the Committee on Publication cause two copies of the *Transactions* of the Association to be transmitted to the British Association, through the British legation at Washington, with a request for an interchange of *Transactions.*

Dr. Peter Parker, ex-commissioner to China, was then introduced, and was received with applause. He exhibited some curious specimens of *calculi,* as the results of thirty-eight operations upon Chinese. They were of various shapes and composition, and weighed from a few drachms up to three, seven, and eight ounces. His description of the operation by which these calculi were removed was deeply interesting, and it was gratifying to learn, that out of the thirty-eight patients all but five or six recovered perfect health.

Dr. Parker proceeded to state, that he has treated in China, at the hospital under his charge, fifty-three thousand cases. Pictures of the most curious cases he had brought to this country, and they were on exhibition in the room below. At no very distant period he hopes to place in a permanent form the result of his labors, with illustrations. Among other cases, he had probably performed upwards of a thousand operations for cataract. On one day he operated in sixteen cases, the youngest being a mere child, and the oldest an old lady seventy-nine years of age. She came, led by a servant, submitted heroically to operations on both eyes the same day, and in a fortnight had her sight perfectly restored.

Dr. Dunbar, of Maryland, then offered the following resolution, which was adopted:

*Resolved,* That the thanks of the American Medical Association are due to Dr. Peter Parker, for the very interesting report that he has made of his surgical operations in China, and his specimens which he has exhibited ; and when his work shall be published, that the members of this Association be recommended to patronize the same, by obtaining copies thereof.

In acknowledging this vote of thanks, Dr. Parker said he had among his patients all classes, from members of the imperial family down to beggars. His greatest difficulty had been to persuade his patients that he could not cure all diseases.

On motion of Dr. Anderson, of New York, it was unanimously

*Resolved,* That the thanks of the Medical Association be presented to Rev. Dr. McGuire and the Faculty of the College of Georgetown, for their very cordial reception and entertainment of the Association at the College.

The Association then, on motion, adjourned until 5 o'clock, P. M.

---

*Afternoon Session.*—The Association was called to order at 5 o'clock, P. M., by Dr. W. L. Sutton, one of the Vice-Presidents, who took the chair.

Dr. T. L. Mason, of New York, moved to amend the Constitution as follows:

In first line, second paragraph of Article 2, after the words, "shall receive the appointment from," to insert, "any medical society *permanently* organized in accordance with the laws regulating the practice of physic and surgery in the state in which they are situated; and consisting of physicians and surgeons regularly authorized to practice their profession." Laid over to next year, under the rules.

To add to the sixth paragraph, Article 2, "But each permanent member of the first class designated in this place of organization, shall be entitled to a seat in this Association only on his presenting to this body a certificate of his good standing, signed by the secretary of the society to which he may belong at the time of each annual meeting of this body." Laid over to next year, under the rules.

Dr. Henry Hartshorne, of Pennsylvania, moved the following amendment to the Constitution, which was also laid over, viz.

To add to the 2d Article: "No one expelled from this Association shall, at any time thereafter, be received as delegate or member, unless by a three-fourths vote of the members present at the meeting to which he is sent, or to which he is proposed.

Dr. John Watson, of New York, from the Committee on Ethics, reported as follows:

*Whereas*, it appears from undoubted testimony that the New York Medical College have conferred the degree of Doctor of Medicine upon a notorious quack of the name of John F. Dunker, of Newark, the Faculty, in the person of the President of said College, wish here to declare that this degree was obtained under gross deception, and false testimonials furnished by said Dunker and his friends, and they therefore revoke and annul his diploma, and declare said Dunker to be unworthy of patronage or support, from authority conferred upon him by this diploma.

Dr. C. C. Cox, of Maryland, moved that the report be indefinitely postponed, which was not agreed to.

On motion, the report was then adopted.

The amendments proposed by Dr. Stocker, of Pennsylvania, at the annual meeting in 1856, at Detroit, were then taken from the table for consideration, upon motion.

The following were the amendments proposed, viz.

*Article* 3. Strike out all after the words "first Tuesday in May," and insert as follows: "The Association shall meet biennially in the city of ———. The place of meeting for the intermediate year shall be determined by a vote of the Association."

*Article* 4. In first paragraph, second line, instead of the words "two Secretaries," insert "one permanent and two assistant Secretaries." In the fourth paragraph, fifth line, strike out the words "the Secretary," &c., and substitute "The *permanent* Secretary shall preserve the archives and unpublished transactions in the permanent place of meeting of the Association. His expenses for traveling to and from the place of meeting, and while in attendance upon the same, shall be defrayed by the Association."

Upon the amendment proposed to Article 3, the vote was, yeas 80, nays 42. And upon the first amendment proposed to Article 4, the vote was, yeas 84, nays 53. Neither of the amendments having received the constitutional majority of two-thirds, the President declared them to be rejected.

A motion was made to postpone indefinitely the second amendment proposed to Article 4.

Dr. Stocker moved to lay the motion to postpone upon the table.

Pending this, a motion was made to lay the amendment upon the table, which was agreed to.

Dr. Robt. C. Foster, of Tennessee, offered the following resolution, which was adopted, viz.

*Resolved*, That the Committee on Publication be instructed to collect all of the by-laws and resolutions which have not been rescinded through the different volumes of the *Transactions;* to arrange them under their respective heads, and append the same to the Constitution.

The following amendment to the Constitution was proposed by Dr. J. Berrien Lindsley, of Tennessee, through Dr. Bowling, of Tennessee, and was laid over under the rules, viz.

"In Article 2, omit the words 'medical colleges, hospitals, lunatic asylums, and other permanently organized medical institutions of good standing in the United States;' and also omit the words: 'The Faculty of every regular constituted medical college or chartered school of medicine, shall have the privilege of sending two delegates. The professional staff of every chartered or municipal hospital containing an hundred inmates or more, shall have the privilege of sending two delegates, and every other permanently organized medical institution of good standing shall have the privilege of sending one delegate."

The President announced that the members of the Association were invited by the Curator of the Washington Infirmary to visit that Institution.

Upon motion of Dr. Kemp, of Maryland, the thanks of the Association were extended to those railroad companies which had agreed to pass the members to their home free of charge; and also to all those citizens of Washington, including members of the press, whose courteous attentions had placed the Association under obligations.

On motion, the Association then adjourned *sine die.*

